# Supplementary material for: The Cytokine, Chemokine, and Growth Factor Network of Prenatal Depression
Source: Brain Sci. 2023 Apr 26;13(5):727. doi: 10.3390/brainsci13050727 (PMC10216549; doi:10.3390/brainsci13050727)
Supplement: Supplementary file 1 [file brainsci-13-00727-s001.zip › brainsci-2344874-supplementary.pdf]

The Cytokine, Chemokine, and Growth Factor Network of Prenatal Depression

Michael Maes [1,2,3,4,5,*], Yoshiko Abe [6,7], Wandee Sirichokchatchawan [6,8], Junpen Suwimonteerabutr [9], Ussanee Sangkomkamhangd [10], Abbas F. Almulla [1,11] and Sirina Satthapisit [12]

[1] Department of Psychiatry, Faculty of Medicine, Chulalongkorn University and King Chulalongkorn Memorial Hospital, the Thai Red Cross Society, Bangkok 10330, Thailand
[2] Kyung Hee University, 26 Kyungheedae-ro, Dongdaemun-gu, Seoul 02447, Republic of Korea
[3] Department of Psychiatry, Medical University of Plovdiv, 4002 Plovdiv, Bulgaria
[4] Research Institute, Medical University of Plovdiv, 4002 Plovdiv, Bulgaria
[5] IMPACT Strategic Research Center, Barwon Health, Geelong 3220, Australia
[6] College of Public Health Sciences (CPHS), Chulalongkorn University, Bangkok 10330, Thailand
[7] Department of Public Health, Dokkyo Medical University School of Medicine, Tochigi 321-0293, Japan
[8] Health and Social Sciences and Addiction Research Unit, Chulalongkorn University, Bangkok 10330, Thailand
[9] Department of Obstetrics, Gynaecology and Reproduction, Faculty of Veterinary Science, Chulalongkorn University, Bangkok 10330, Thailand
[10] Department of Obstetrics and Gynecology, Khon Kaen Hospital, Khon Kaen 40000, Thailand
[11] Medical Laboratory Technology Department, College of Medical Technology, The Islamic University, Najaf 54001, Iraq
[12] Department of Psychiatry, Khon Kaen Hospital, Khon Kaen 40000, Thailand
* Correspondence: dr.michaelmaes@hotmail.com

ESF, Table S1. Cytokines, chemokines and growth factors measured in the current study

Protein abbreviations	Gene ID	Protein name	Cases with values > lower limit OOR
IL-1β	IL1B	Interleukin-1β	33.3%
IL-1RA	IL1RN	Interleukin-1 receptor antagonist	87.5%
IL-2	IL2	Interleukin-2	8.8%
IL-4	IL4	Interleukin-4	69.9%
IL-5	IL5	Interleukin-5	0%
IL-6	IL6	Interleukin-6	42.3%
IL-7	IL7	Interleukin-7	28.7%
CXCL8 / IL-8	CXCL8	C-X-C motif chemokine ligand 8	89.8%
IL-9	IL9	Interleukin-9	99.5%
IL-10	IL10	Interleukin-10	19.4%
IL-12p70	IL12	Interleukin-12	56.9%
IL-13	IL13	Interleukin-13	65.7%
IL-15	IL15	Interleukin-15	42.1%
IL-17	IL17	Interleukin-17	40.7%
CCL11	CCL11	Eotaxin	99.5%
FGF2	FGF2	Fibroblast growth factor 2, Basic fibroblast growth factor	95.8%
G-CSF	CSF3	Granulocyte Colony Stimulating Factor, Colony Stimulating Factor 3 (Granulocyte)	96.3%
GM-CSF	CSF2	Granulocyte-macrophage colony-stimulating factor, Colony-stimulating factor 2	63.0%
IFN-γ	IFNG	Interferon-γ	38.0%
CXCL10 /IP-10	CXCL10	C-X-C motif chemokine ligand 8, Interferon gamma-induced protein 10	100%
CCL2 / MCP1	CCL2	C-C Motif Chemokine Ligand 2	99.5%

CCL3 / MIP-1α	CCL3	Macrophage inflammatory protein-1 alpha, C-C Motif Chemokine Ligand 3	97.0%
PDGF	PDGFB	Platelet Derived Growth Factor Subunit B	99.5%
CCL4 / MIP-1β	CCL4	C-C Motif Chemokine Ligand 4, Macrophage Inflammatory Protein 1-Beta, Lymphocyte Activation Gene 1 Protein	99.5%
CCL5 /RANTES	CCL5	C-C Motif Chemokine Ligand 5, Regulated Upon Activation, Normally T-Expressed, And Presumably Secreted	30.1%
TNF-α	TNF	Tumor Necrosis Factor-Alpha	99.1%
VEGF	VEGFA	Vascular Endothelial Growth Factor	21.3%

ESF, Table S2. Computation of the immune profile composite scores assessed in this study

**Immune Profile**	**Members**
**M1 macrophage**	IL-1β, sIL-1RA, IL-6, TNF-α, CXCL8, CCL3
**T helper-1**	IL-2, IFN-γ, IL-12
**T helper-2**	IL-4, IL-5, IL-9, IL-13
**T helper-17**	IL-6, IL-17
**IRS**	IL-1β, IL-6, TNF-α, CXCL8, CCL3, IL-2, IFN-γ, IL-12, IL-17, IL-15, G-CSF, GM-CSF, CXCL10, CCL5, CCL2
**CIRS**	IL-4, IL-10, sIL-1RA
**Growth factors**	FGF, PDGF, VEGF
**Chemokines**	CXCL8, CXCL10, CCL2, CCL3, CCL4, CCL5, CCL11
**T cell growth**	IL4, IL9, IL12, GM-CSF, IL-15
**IRS/CIRS**	(All IRS) / (All CIRS)

IRS: immune-inflammatory response system; CIRS: compensatory immunoregulatory system

ESF2, Table S3. Results of univariate GLM analyses of the immune profiles and the two-step-cluster analysis-generated classes with age and BMI as covariates.

Estimates

Dependent Variable	Clusters	Mean	Std. Error	95% Confidence Interval	
				Lower Bound	Upper Bound
M1	Cluster 1	-.159[a]	.110	-.377	.059
	Cluster 2	.275[a]	.145	-.012	.562
Th1	Cluster 1	-.226[a]	.106	-.437	-.015
	Cluster 2	.390[a]	.140	.114	.667
Th2	Cluster 1	-.160[a]	.113	-.384	.064
	Cluster 2	.276[a]	.149	-.019	.570
Th17	Cluster 1	-.184[a]	.110	-.401	.033
	Cluster 2	.318[a]	.144	.032	.603
IRS	Cluster 1	-.216[a]	.105	-.424	-.008
	Cluster 2	.373[a]	.138	.100	.646
CIRS	Cluster 1	-.118[a]	.110	-.336	.100
	Cluster 2	.204[a]	.145	-.082	.490
GF	Cluster 1	-.162[a]	.108	-.377	.052
	Cluster 2	.280[a]	.142	-.001	.562
Chemokines	Cluster 1	-.150[a]	.107	-.362	.062
	Cluster 2	.259[a]	.141	-.020	.538
TcellG	Cluster 1	-.224[a]	.109	-.439	-.009
	Cluster 2	.387[a]	.143	.104	.670
IRS/CIRS	Cluster 1	-.157[a]	.113	-.382	.067
	Cluster 2	.271[a]	.149	-.024	.566

a. Covariates appearing in the model are evaluated at the following values: Age = 27.21, BMI = 23.320.

Univariate Tests

Dependent Variable		Sum of Squares	df	Mean Square	F	Sig.
M1	Contrast	5.241	1	5.241	5.694	.019
	Error	106.772	116	.920		
Th1	Contrast	10.561	1	10.561	12.297	<.001
	Error	99.625	116	.859		
Th2	Contrast	5.269	1	5.269	5.422	.022
	Error	112.731	116	.972		
Th17	Contrast	6.989	1	6.989	7.652	.007
	Error	105.948	116	.913		
IRS	Contrast	9.637	1	9.637	11.537	<.001
	Error	96.898	116	.835		
CIRS	Contrast	2.882	1	2.882	3.138	.079
	Error	106.534	116	.918		
GF	Contrast	5.443	1	5.443	6.128	.015
	Error	103.022	116	.888		
Chemokines	Contrast	4.647	1	4.647	5.324	.023
	Error	101.253	116	.873		
TcellG	Contrast	10.368	1	10.368	11.590	<.001
	Error	103.769	116	.895		
IRS/CIRS	Contrast	5.097	1	5.097	5.224	.024

Error	113.177	116	.976		

The F tests the effect of Clusters. This test is based on the linearly independent pairwise comparisons among the estimated marginal means.

ESF, Table S4. Results of univariate GLM analyses of the single cytokines and growth factors and the two-step-cluster (TSC)-analysis-generated classes with age and BMI as covariates.

Estimates

				95% Confidence Interval	
Dependent Variable	Clusters	Mean	Std. Error	Lower Bound	Upper Bound
sIL1RA	Cluster 1	-.087[a]	.113	-.310	.136
	Cluster 2	.150[a]	.148	-.143	.443
IL4	Cluster 1	-.168[a]	.113	-.392	.056
	Cluster 2	.290[a]	.149	-.005	.585
IL6	Cluster 1	-.143[a]	.110	-.360	.075
	Cluster 2	.247[a]	.144	-.039	.532
IL8	Cluster 1	-.150[a]	.113	-.374	.074
	Cluster 2	.259[a]	.149	-.035	.554
IL9	Cluster 1	-.055[a]	.113	-.279	.169
	Cluster 2	.095[a]	.149	-.199	.390
IL12	Cluster 1	-.182[a]	.109	-.398	.034
	Cluster 2	.314[a]	.143	.030	.598
IL13	Cluster 1	-.112[a]	.115	-.339	.115
	Cluster 2	.194[a]	.151	-.105	.492
IL15	Cluster 1	-.150[a]	.110	-.368	.067
	Cluster 2	.260[a]	.144	-.026	.546
IL17	Cluster 1	-.141[a]	.114	-.366	.084
	Cluster 2	.244[a]	.150	-.053	.540
TNFa	Cluster 1	-.094[a]	.115	-.321	.134
	Cluster 2	.162[a]	.151	-.137	.461

G_CSF	Cluster 1	-.109[a]	.111	-.328	.110
	Cluster 2	.188[a]	.145	-.100	.476
GM_CSF	Cluster 1	-.163[a]	.110	-.382	.055
	Cluster 2	.282[a]	.145	-.006	.570
MIP1A	Cluster 1	-.092[a]	.113	-.316	.132
	Cluster 2	.159[a]	.149	-.135	.454
MIP1b	Cluster 1	-.124[a]	.110	-.341	.094
	Cluster 2	.213[a]	.145	-.073	.500
MCP1)	Cluster 1	-.092[a]	.112	-.314	.130
	Cluster 2	.159[a]	.147	-.133	.451
CXCL10	Cluster 1	-.089[a]	.112	-.310	.132
	Cluster 2	.154[a]	.147	-.137	.445
CCL11	Cluster 1	-.085[a]	.115	-.313	.144
	Cluster 2	.146[a]	.151	-.154	.446
FGF	Cluster 1	-.091[a]	.114	-.317	.135
	Cluster 2	.157[a]	.150	-.140	.454
PDGF	Cluster 1	-.062[a]	.110	-.280	.156
	Cluster 2	.107[a]	.145	-.179	.394

a. Covariates appearing in the model are evaluated at the following values: Age = 27.21, BMI = 23.320.

Univariate Tests

Dependent Variable		Sum of Squares	df	Mean Square	F	Sig.
sIL1RA	Contrast	1.562	1	1.562	1.625	.205
	Error	111.561	116	.962		

IL4	Contrast	5.831	1	5.831	5.980	.016
	Error	113.099	116	.975		
IL6	Contrast	4.209	1	4.209	4.606	.034
	Error	105.988	116	.914		
IL8	Contrast	4.656	1	4.656	4.798	.030
	Error	112.585	116	.971		
IL9	Contrast	.631	1	.631	.649	.422
	Error	112.635	116	.971		
IL12	Contrast	6.834	1	6.834	7.581	.007
	Error	104.575	116	.902		
IL13	Contrast	2.594	1	2.594	2.598	.110
	Error	115.847	116	.999		
IL15	Contrast	4.678	1	4.678	5.121	.025
	Error	105.968	116	.914		
IL17	Contrast	4.112	1	4.112	4.184	.043
	Error	114.004	116	.983		
TNFa	Contrast	1.820	1	1.820	1.818	.180
	Error	116.114	116	1.001		
G_CSF	Contrast	2.439	1	2.439	2.626	.108
	Error	107.722	116	.929		
GM_CSF	Contrast	5.509	1	5.509	5.949	.016
	Error	107.429	116	.926		
MIP1A	Contrast	1.755	1	1.755	1.810	.181
	Error	112.498	116	.970		
MIP1b	Contrast	3.156	1	3.156	3.440	.066
	Error	106.438	116	.918		

MCP1)	Contrast	1.750	1	1.750	1.833	.178
	Error	110.754	116	.955		
CXCL10	Contrast	1.641	1	1.641	1.736	.190
	Error	109.671	116	.945		
CCL11	Contrast	1.476	1	1.476	1.466	.228
	Error	116.742	116	1.006		
FGF	Contrast	1.708	1	1.708	1.734	.190
	Error	114.252	116	.985		
PDGF	Contrast	.796	1	.796	.866	.354
	Error	106.582	116	.919		

The F tests the effect of Clusters. This test is based on the linearly independent pairwise comparisons among the estimated marginal means.

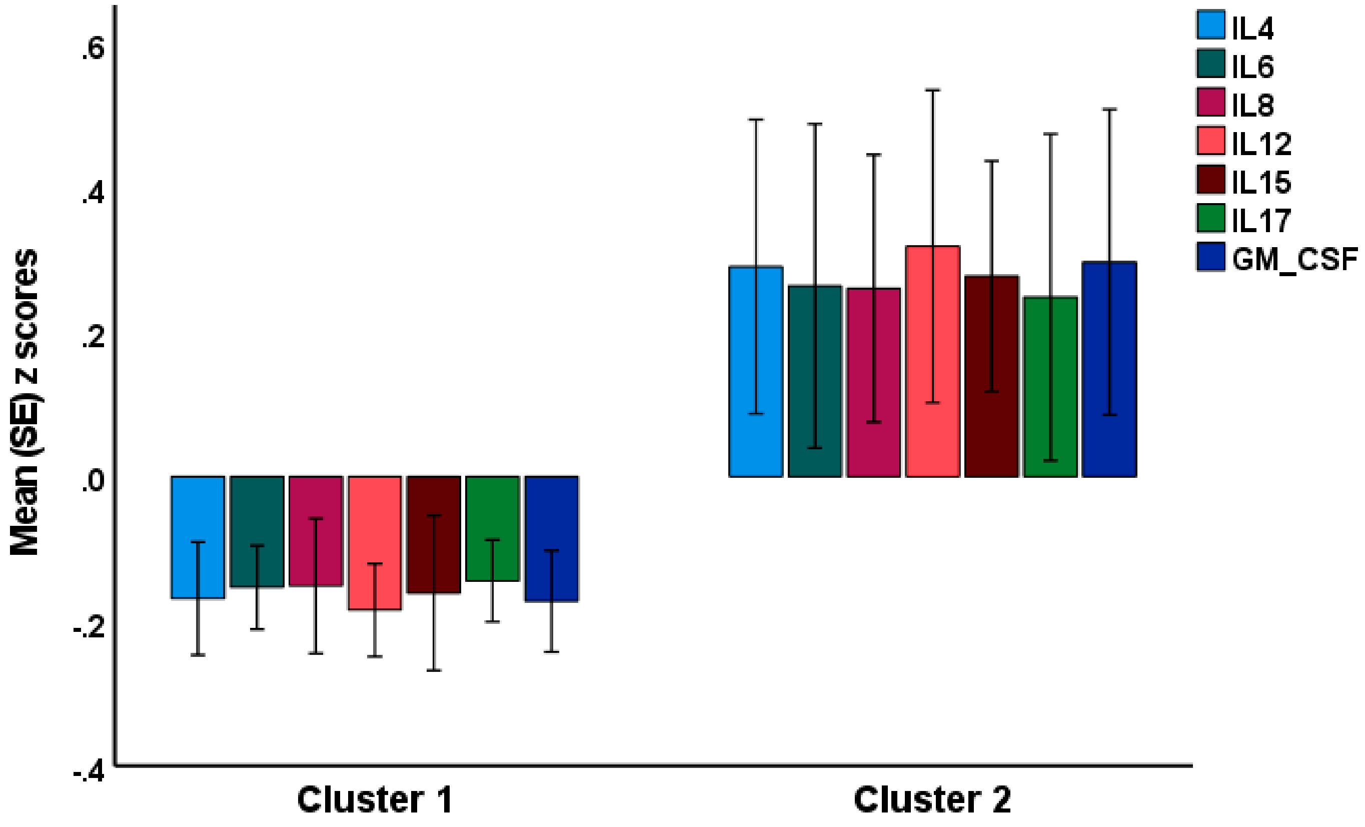

ESF, Figure S1. Results of cluster analysis performed on the Edinburgh Postnatal Depression Scale scores and immune profiles.IL: interleukin; GM-CSF: granulocyte-macrophage colony stimulating factor

ESF, Table S5. Results of general estimation equations (repeated measurements) analyses with the early and late Edinburgh Postnatal Depression Scale (EPDS) scores and immune profiles as dependent variables and time (early-late pregnancy phase), the cluster analysis-generated classes, and their interaction as explanatory variables.

Variables	Time, early	Time, late	Source	Wald (df=1)	P
EPDS Cluster 1	3.42 (0.29)	4.34 (0.36)	Time	18.17	<0.001
EPDS Cluster 2	10.41 (0.67)	6.29 (0.69)	Clusters	43.28	<0.001
			Interaction	44.93	<0.001
M1	-0.077 (0.901)	0.205 (0.093)	Time	6.48	0.011
Th1 Cluster 1	-0.295 (0.081)	0.0237 (0.122)	Time	0.224	0.636
Th1 Cluster 2	0.356 (0.198)	0.152 (0.164)	Clusters	5.23	0.022
			Interactions	4.64	0.031
Th-17 Cluster 1	-0.310 (0.062)	0.116 (0.108)	Interaction	4.72	0.030
Th-17 Cluster 2	0.255 (0.234)	0.155 (0.160)			
IRS Cluster 1	-0.361 (0.069)	0.136 (0.114)	Clusters	4.04	0.044
IRS Cluster 2	0.263 (0.208)	0.220 (0.181)	Interaction	4.61	0.031
CIRS	0.216 (0.106)	-0.200 (0.113)	Time	9.21	0.002
GF Cluster 1	-0.298 (0.093)	0.252 (0.125)	Interaction	5.80	0.016
GF Cluster 2	0.139 (0.153)	0.041 (0.202)			
Tcell growth Cluster 1	-0.291 (0.700)	0.193 (0.125)	Clusters	4.66	0.031
Tcell growth Cluster 2	0.292 (0.184)	0.233 (0.206)			
IRS/CIRS	-0.373 (0.093)	0.534 (0.091)	Time	69.90	<0.001

Shown are the significant sources of variance. EPDS: Edinburgh Postnatal Depression Scale; M1: macrophage M1, Th: T helper, IRS: immune-inflammatory response system, CIRS: compensatory immunoregulatory system, GF: growth factors.
